# Effect of Silicon Fertilization on Crop Yield Quantity and Quality—A Literature Review in Europe

**DOI:** 10.3390/plants7030054

**Published:** 2018-07-06

**Authors:** Arkadiusz Artyszak

**Affiliations:** Department of Agronomy, Warsaw University of Life Sciences, 159 Nowoursynowska St., 02-776 Warsaw, Poland; arkadiusz_artyszak@sggw.pl; Tel.: +48-22-59-32-702

**Keywords:** silicon, yield, crop quality, fertilization

## Abstract

This paper presents a research review of the effect of silicon fertilization on the yield quantity and quality in the last 15 years. The study focuses on plant species grown in Europe: cereals, soybean, rapeseed, sugar beet, potato, meadows, berries and vegetables, and orchard and ornamental plants. The use of silicon is most common in the production of vegetables in greenhouses. However, the use of this element for the fertilization of agricultural plants is rare. Positive prospects of silicon fertilization are associated with foliar application, which is much cheaper and more convenient to use than soil fertilization. Foliar application of silicon has a biostimulative effect, and the best results are observed in stressful conditions for plants such as salinity, deficiency or excess of water, high and low temperature, and the strong pressure of diseases and pests, etc. Based on the results of previous studies, it can be concluded that foliar nutrition should be introduced into production as a standard treatment in the crop management of many species of agricultural plants. It can help farmers to increase the yield of crops. It is also important that it is safe for the environment, which is particularly important in Europe.

## 1. Introduction

Silicon (Si) is classified as a beneficial element. This element limits the effects of abiotic and biotic stresses in plants. The most known effect is the effect of silicon on plants, which uptake the largest amounts of this element, i.e., in sugar cane and rice. However, these species are rarely grown in Europe, and, moreover, much less research on this aspect has been conducted on plants that have the greatest economic importance in Europe: cereals, rapeseed, sugar beet, and potato.

In recent years, more research has been performed with regards to foliar nutrition using silicon, which brings unequivocal production benefits, and at the same time is much cheaper and more convenient to use than soil fertilization. As a result, it improves the profitability of many plant species’ production. Hopes are high regarding its effect on the reduction of drought stress, which is increasingly affecting agriculture in Europe. The positive effect of silicon observed, in the studies, results in many cases in which the susceptibility of plants to drought stress is reduced.

Silicon has also a beneficial effect on limiting the adverse effects of other abiotic stresses, caused by salinity, heavy metals, high and low temperature, water flooding, etc. Taking account of all these factors, the most important for Europe are too high and too low temperatures, but they only concern certain areas of the continent (mainly too low temperature). The significance of other abiotic factors is relatively low in Europe.

High hopes are related to the use of silicon to limit the effects of biotic stress caused by diseases and pests. Obtaining improvement of yield and crop quality in many experiments was often the result of increased resistance of plants to infection by diseases caused mainly by fungi. The use of this element for foliar nutrition of plants may become an important element of integrated plant protection in the near future, which all farmers in the European Union are obliged to apply. Also important is the fact that foliar application of silicon is safe for the natural environment and can also be used in organic farming, which is becoming more important in Europe.

The increase of yield observed in many studies, after applying foliar nutrition with silicon, was caused by the improvement of all or some of the yield components. In the case of most common cereals, they were: a spike density during harvest, a number of kernels per spike, and a mass of 1000 grains [1,2]. An increase in the number of grains per cob and their mass was observed in maize [3]. A higher yield of soybean caused by foliar application of silicon resulted from the increase in the number of pods per plant, the number of seeds in the pod, and the weight of 1000 seeds [4]. In the cultivation of winter oilseed rape, the improvement of plants’ resistance to winter conditions was observed, as well as the formation of larger seeds [5,6]. In the case of sugar beet, foliar nutrition with silicon contributed to the increase of fresh root mass, and increase of the root yield, which determines the yield of sugar [5,7,8,9,10,11,12]. In the potato cultivation, plants fertilized with silicon developed tubers with a larger fresh mass [13,14,15].

## 2. Cereals

Evaluation of the effect of foliar application of silicon on wheat production was conducted in Iran [16]. Foliar application of 6 mM sodium silicate at various stages of wheat growth provided higher resistance to drought by maintaining cellular membrane integrity, relative water content, and increasing chlorophyll content. The highest positive influence of silicon application was observed with the use of silicon both at the tillering and anthesis stages.

Another study in which the effect of silicon was examined on wheat production was conducted in Idaho [17]. Three concentrations of Si, i.e., 140, 280, and 560 kg Si∙ha^−1^ for fertilization, were applied in the form of amorphous volcanic tuff. The results did not significantly prove the effect of Si application on grain yield or grain protein content of irrigated winter wheat grown in nonstressed conditions.

In a field study conducted in Louisiana, effect of five silicate slag concentrations (0, 1, 2, 4.5, and 9 Mg∙ha^−1^) on productivity of wheat under sufficient and high nitrogen (N) application concentrations was examined [1]. Si application caused increase of grain yield, number of spikes per square meter, and number of grains per spike. Positive effect of silicate slag application was especially observed in wheat fertilized with high N, and in soil with low Si plant-availability.

In a pot experiment conducted in Egypt, it was proved that foliar fertilization of winter wheat with silicon in the form of sodium silicate (250 and 1000 mg SiO_2_∙kg^−1^) or silicon with boron (25 mg B∙kg^−1^) in both saline and nonsaline soil, applied twice (40 and 70 days after sowing), significantly increased the height of shoots and the leaf surface, as well as the grain yield per plant and the weight of 1000 grains in relation to the control [2]. In this experiment, the treatment with silicon mitigated negative effect of soil salinity on the plant growth, grain yield, and uptake of nutrients. In the field conditions, the best effect was observed when the lowest silicon dose (250 mg SiO_2_∙kg^−1^) was applied.

In India, the positive effect of soil fertilization with silicon (in the form of calcium silicate) and phosphorus on the improvement of lodging resistance of wheat and increase in yield [18] was confirmed. In several experiments with wheat in Mexico, foliar application of silicon twice, as well as soil application of Armurox (a complex of peptides with soluble silicon), had a positive effect on increasing plant resistance to lodging [19].

In Albania, foliar application of marine calcite (Herbagreen) to wheat with a reduced (30%) application of NPK fertilizers yielded grains with the same protein and fat content as at the full NPK dose [20]. In the opinion of these authors, it may have a beneficial effect on the reduction of soil and water pollution caused by the standard application of mineral fertilizers, and also be profitable for farmers using such application. In the United States, beneficial effects of applying silicon (140, 280, and 560 kg Si∙ha^−1^) to the soil were observed in the cultivation of winter wheat irrigated throughout the growing season [21]. Also, foliar fertilization with silicon significantly influenced the increase of wheat yield in studies conducted in Pakistan [22] and Egypt [23].

In Germany, the production of aboveground biomass of wheat was enhanced by silicon fertilization. The highest increase of biomass was found at low Si supply. The rise in biomass production was mainly caused by a substantial increase of straw biomass [24]. In the study, grain yield only increased significantly at medium level of supply of silicon (10 g SiO_2_∙pot^−1^), probably due to increased plant phosphorus availability and nutrition.

Another study looked at the effect of silicon (Si) application under drought stress on wheat. Silicon potassium metasilicate (K_2_SiO_3_) was applied at the rate of 0 and 12 kg∙ha^−1^. Concentration of K^+^ in shoot and grains increased in Si application, which allowed one to maintain water potential in plant and caused higher yield and biomass [25].

In the study conducted during two growing seasons in Brazil, evaluation of changes caused in the wheat crop yield components by silicon foliar application was conducted. Effect of three doses of silicon (0, 3, and 6 dm^3^ of Si∙ha^−1^), which were applied during tillering, booting, and anthesis stages, was examined. The harvest index (first year) and the number of tillers (second year), however, presented a relationship with the supply of silicon. The remaining differences were attributed to variations among the wheat cultivars [26].

Another study, conducted in Brazil, examined the result of foliar fertilization with silicon in wheat and its effect on seed yield and physiological quality. Treatments consisted of two silicon dosages (three and six liters silicon per hectare) and the control (no silicon) and five wheat cultivars. The results showed that the foliar application of silicon at the dosages tested did not affect the yield and physiological quality of the seeds produced by the wheat cultivars [27].

In India, in the years 2016–2017, a significant increase in corn grain yield and improvement of grain quality was observed when silicon in the form of monosilicic acid was applied to the soil (Silixol granules) and foliar fertilization (Silixol plus) [28] was applied. These authors, as the most profitable option, recommended the use of standard fertilization (NPK) for corn + 25 kg of Silixol granules∙ha^−1^ to the soil and foliar application of Silixol plus at a concentration of 1 cm^3^∙dm^−3^. Foliar application of AB Yellow (microcolloidal silicic acid with 2% micronutrients: B—0.3%, Cu—0.15%, Mo—0.1%, and Zn—1.5%) in wheat in Romania in 2014 on very saline soil resulted in increase in grain yield by 340% [29].

In Poland, under laboratory conditions, the effect of Optysil (94 g Si∙dm^−3^), a growth stimulator, was studied to reduce the negative impact of drought stress on wheat [30]. Lab tests on wheat showed a 41% lower leakage of electrolytes and an increase in protein production by 40% compared to the control combination.

In the study conducted in Pakistan, effect of Si application on maize was examined in pot experiment. In drought-stressed plants of hybrid P-33H25 and FH-810 silicon application significantly increased plant height, stem diameter, number of leaves, cob length, number of grains/cob, 100 grain weight, grain yield, and biological yield. Silicon application to drought stressed maize plants improved the growth and yield, which are caused by higher photosynthetic rate and lowered transpiration [3].

In the Department of Mycology of the Institute of Plant Protection—NRI in Poznań (Poland), the effect of foliar application of the Optysil stimulator on the content of mycotoxins in corn grain [31] was investigated. In the field experiment, three doses of the stimulator were applied: 0.5, 1, and 2 dm^3^∙ha^−1^. The spraying was performed three times: at BBCH 14, BBCH 17, and BBCH 19–39 stages. In the seed from control plots (nonspray), the deoxynivalenol (DON) content was 277 μg∙kg^−1^ and zearelon (UAE) 173.4 μg∙kg^−1^. In treatments with Optysil, the content of mycotoxins was significantly reduced. DON content was 47.6 μg∙kg^−1^ when 0.5 dm^3^∙ha^−1^ of Optysil was applied, 30.6 μg∙kg^−1^ when a dose of 1 dm^3^∙ha^−1^ was applied, and 25.5 μg∙kg^−1^ when 2 dm^3^∙ha^−1^ was applied. The reduction in ZEA content was higher, and the content of this mycotoxin was 5.8, 1.6, and 2.7 μg∙kg^−1^, respectively. Therefore, the authors recommended foliar spraying with the growth stimulator Optysil as the standard procedure in corn management in Poland.

Studies of silicon application to the soil conducted in north-east China in the years 2005–2006 indicated an increase in corn yield by 5.6–10.4% (on average by 7.7%) in comparison with the control variant [32].

The increase in grain yield of cereal observed in the study in which silicon fertilization was applied was caused by increase in spike density during harvest, the number of grains per spike, and, sometimes, the weight of 1000 grains. The increase in grain yield of maize was caused by the greater number of grains per cob and the weight of 1000 grains.

## 3. Soybean

In studies conducted in India, foliar application of silicic acid (2% soluble H_4_SiO_4_) at a dose of 2 or 4 cm^3^∙dm^−3^ doubled or tripled, and thus significantly improved, soybean growth and yield [33]. For economic reasons, the authors recommended using a smaller dose (2 cm^3^∙dm^−3^) in three sprays.

In one of the experiments performed in Poland, the effect of foliar application of silicon on soybean in a plastic tunnel and in field conditions was studied [4]. These experiments conducted in open plastic tunnels included control (plants watered to 70% of the water capacity of the soil, not sprayed with silicon), drought (up to 25% of the water capacity of the soil from the full flowering period of the plant for 3 weeks), drought + use of Optysil stimulator at a concentration of 0.25% in spraying, and drought + application of the Silvit stimulator (SiO_2_—150 g∙dm^−3^, K_2_O—100 g∙dm^−3^, SO_3_—25 g∙dm^−3^, B—1.25 g∙dm^−3^, Zn—0.25 g∙dm^−3^, and amino acids) at a concentration of 0.25%. The stimulators were sprayed three times in 2-week intervals, starting from the 6-leaf leaf stage of soybean plants. During the cultivation, plants subjected to drought stress aborted over 92% of flowers and young pods, while irrigated plants (control) aborted 85% of flowers and young pods. The applied stimulators had not reduced the number of fallen flowers, but increased the number of pods per plant (Silvit by 20%, and Optysil by 18%) in comparison with plants in drought conditions without stimulators. Silvit increased the number of seeds per plant by 27%, the number of seeds in the pod by 11%, and the yield of seeds per plant by 12% in comparison with the plants in drought conditions without stimulators. In the field experiment, the Optysil stimulator at a concentration of 0.25% in spraying was applied three times, every 2 weeks starting from the six-leaf stage. Such treatment caused an increase in the number of pods by approx. 14% in relation to the control and weight of seeds per plant by 21%. Application of Optysil stimulator also increased weight of 1000 seeds by 9%. In addition, Optysil also increased the number of seeds per plant by 12%.

In another field experiment conducted in Poland, the use of Optysil increased number of pods in soybeans by 18%, and the average seed yield per plant by 21% compared to the control treatment [30].

Studies on application of silicon to the soil conducted in China in the years 2005–2006 indicated an increase in soybean yields by 7.5–13.6% (on average by 11.0%) in comparison with the control treatment [32].

Improvement in soybean yielding due to the use of silicon was caused by increase in the number of pods per plant, the number of seeds in the pod, and the weight of 1000 seeds.

## 4. Rapeseed

The experiment with the foliar application of Optysil growth stimulator in the cultivation of winter oilseed rape in three locations in Poland was performed in the years 2011/2012–2012/2013 [34]. The rapeseed yield increased from 1.7% to 17% depending on the variety of rapeseed and location. At the same time, increase in the weight of 1000 seeds was observed from 1.4 to 19% compared to the control treatment (without foliar fertilization). A similar study in Poland conducted in 2010/2011–2011/2012, on the use of sea calcite (Herbagreen Basic) in autumn (393 g Ca∙ha^−1^ and 120 g Si∙ha^−1^) in the four- to six-leaf growth stage in rapeseed, resulted in significant reduction in the height of the apical bud, which could have contributed to better resistance of plants in winter conditions [5]. In treatments with marine calcite, plant losses during winter were, on average, 5.5%, and in unsprayed conditions they were 15.4%. In 2011, the highest yield of seeds, with the highest fat content and the largest fat yield, was obtained using one autumn spray (393 g Ca∙ha^−1^ and 120 g Si∙ha^−1^) in the four- to six-leaf rapeseed growth stage. In 2012, the most beneficial yield in terms of yield of seeds and fat yield was the one application during the start of vegetation (393 g Ca∙ha^−1^ and 120 g Si∙ha^−1^). The fat content in rapeseeds was similar for all treatments. In studies conducted in Poland, application of the Silvit stimulator at a dose of 0.2 dm^3^∙ha^−1^ in autumn in the two- to four-leaf rapeseed growth stage (BBCH 12–14) increased the number of rosette leaves, root collar diameter, height of the apical bud, and main root length compared to the control [6].

Improvement in the yield of winter rape caused by fertilization with silicon corresponds with better wintering and larger plant density. A beneficial effect was also noted in the case of producing more leaves during the fall (which are related with number of lateral shoots) and stronger development of the root system. Plants of rapeseed treated with silicon also formed larger seeds (with a higher weight of 1000 seeds).

## 5. Sugar Beet

In Morocco, since 2014, 13 field trials have been conducted with soil fertilization using Agrisilica (26% of silicon) fertilizer in sugar beet cultivation [35]. The fertilizer was applied during beet sowing at doses of 150, 200, 250, and 300 kg∙ha^−1^ in addition to standard nitrogen and phosphorus fertilization (potassium was not used). Standard fertilization was the control treatment. Sugar beet yield systematically increased up to 40% with the increase of fertilizer dose. A dose of 250 kg∙ha^−1^ resulted in a significant increase in the sugar yield by 4.8 Mg∙ha^−1^ in relation to the control treatment.

In the years 2010–2012, the root yield of the sugar beet cultivar Britannia increased for two treatments of foliar nutrition with marine calcite (Herbagreen Basic) on average by 21.8% compared to the control treatment (without foliar nutrition) [7]. The first treatment at the four- to six-leaf growth stage of beet was 262 g Ca∙ha^−1^ and 79.9 g Si∙ha^−1^, and 3 weeks later was 524 g Ca∙ha^−1^ and 159.8 g Si∙ha^−1^; the second treatment in the of four to six-leaf stage of beet was 524 g Ca∙ha^−1^ and 159.8 g Si∙ha^−1^, and 3 weeks later was 524 g Ca∙ha^−1^ and 159.8 g Si∙ha^−1^. The increase in leaf yield on average for two treatments with foliar nutrition was 28.7% compared to the control treatment; the biological sugar yield (multiplication of root yield and sugar content) was 24.4%, and the commercial yield (biological yield of sugar adjusted by content of components creating molasses) was 24.8%. These authors did not observe any significant differences in the sugar content, pure sugar, α-amino nitrogen, potassium, and sodium in the roots, or in losses in sugar yield, sugar yield productivity, and alkalinity index compared to the control treatment. Using the same experimental treatments in 2011–2012 in Poland increased the root yield of sugar beet cv. Danuśka KWS of 13.1%, leaf yield of 21.0%, biological sugar yield of 15.5%, and commercial yield of sugar of 17.7% compared to the control treatment was observed [8]. These authors did not observe any significant differences in the sugar content in the roots and found a beneficial effect of sea calcite applied as foliar nutrition on the content of pure sugar, and a 22.2% reduction (on average for both foliar nutrition options) of the content of α-amino nitrogen in the roots of sugar beet in comparison to the control treatment. The roots from all treatments did not differ significantly according to the content of potassium and sodium. Application of foliar calcite (Herbagreen Basic) in the years 2013–2014 in Poland in sugar beet Danuśka KWS variety resulted in an average increase of root yield by 12.6% for the three experimental treatments compared to the control treatment (without foliar nutrition), and for using the Optysil growth stimulator by 14.5% (also for three treatments) [9]. None of the treatments with foliar nutrition had a significant impact on yield of leaves compared to the control treatment. The foliar application of marine calcite caused an increase in the biological yield of sugar on average by 14.3%, and the commercial sugar yield by 14.5%, in comparison with the control treatment. In the case of growth stimulator Optysil, it was 14.5% and 14.1%, respectively. Foliar nutrition did not have a significant impact on sugar content and pure sugar compared to the control treatment. However, these authors observed significant differences between the various treatments of foliar nutrition. There was also a lack of significant effect of the application of sea calcite on the content of α-amino nitrogen. However, only in one of the three treatments—the application of Optysil growth stimulator—these authors found a significant increase in the content of α-amino nitrogen (which is the component, which increases molasses content) in comparison to the control treatment. Foliar nutrition also had no significant effect on the potassium content in the roots. However, these authors observed significant differences between the various treatments of foliar nutrition. They also observed a tendency towards reduced sodium content in the roots compared to the control treatment, and in one treatment, the effect of sea calcite was significant.

In a study conducted in 2014 on two production fields in central and eastern Poland, the four-time application of Herbagreen Z20 calcite at a dose of 1 kg∙ha^−1^ did not have any significant impact on the yield of sugar beet roots of the cv. Jagusia compared to the control treatment (without foliar nutrition) [5]. These authors observed an increase in the biological yield of sugar by 2.7% in comparison to the control treatment (11.2 Mg∙ha^−1^), and commercial sugar yield by 4.9% (control—9.60 Mg∙ha^−1^), but these increases were not significant. This was the result of a significant increase in the sugar content in the roots by 3% compared to the control treatment—without foliar nutrition (16.9%). There was also a significant reduction (by 20.2%) in the content of α-amino nitrogen in the roots in comparison to the control treatment (25.8 mmol∙kg^−1^), as well as a significant reduction in the potassium content (by 11.8%) in the roots compared to the control combination (50.7 mmol∙kg^−1^). A decrease in the sodium content of 15.7% was also observed (control—4.45 mmol∙kg^−1^), but this difference was not significant. In studies conducted in Poland in 2016, one foliar application of Agriker Silicium (SiO_2_—207 g∙dm^−3^ and K_2_O—85 g∙dm^−3^) at a dose of 0.5 dm^3^∙ha^−1^ in the six-leaf stage of beet yield increased root yield of the Primadonna KWS variety by 10.6% compared to the control treatment—without foliar nutrition [10]. The additional second spray (Agriker Silicium—0.5 dm^3^∙ha^−1^) 7 days later increased yield by 11.1%, and the third spraying 14 days later by 17.3%. A significant increase was recorded in treatment with three applications. A one-time spray caused increase of yield of leaves by 10.7% compared to the control treatment, two-times spray caused increase by 13.2%, and three-times spray caused increase by 19.1%. Significant increase was found in the treatment with three sprays. The total yield of roots and leaves increased after one application by 10.6%, after two applications by 11.9%, and after three applications by 17.9%. Significant increase was recorded in the treatments with two and three applications. Single foliar application of Agriker Silicium resulted in an increase in the biological sugar yield by 8.5% compared to the control treatment. The additional second spray 7 days later increased yield by 10.9%, and the third spraying 14 days later increased yield by 17.8%. A significant increase was recorded in the treatment with three applications. The increase in commercial sugar yield was 7.8%, 10.4% and 17.4%, respectively. A significant increase was recorded in the treatment with three applications. There were no significant differences in the sugar content, α-amino nitrogen, potassium, sodium, pure sugar, loss of sugar yield, standard loss of molasses, and sugar yield in the roots among all treatments. Single foliar application of Agriker Silicium fertilizer increased the alkalinity factor by 11.0% compared to the control treatment (without foliar nutrition). The additional second spray 7 days later increased the coefficient by 19.5%, and the third spraying 14 days later by 9.8%. Significant increase was recorded in treatments after two applications. In the experiment conducted in Poland in 2015–2016, the effect of foliar nutrition of sugar beet cv. Primadonna KWS with silicon on the yield and commercial quality of roots was assessed [11]. Actisil (6 g Si∙dm^−3^), Herbagreen Z20 (130 g Si∙kg^−1^), and Optysil (94 g Si∙dm^−3^) were applied once (in the six-leaf phase of beet—BBCH 16), twice (BBCH 16 and 7 days later), and three times (BBCH 16, 7 and 14 days later). The dose of Actisil and Optysil was 0.5 dm^3^∙ha^−1^ and Herbagreen Z20 1 kg∙ha^−1^ in 250 dm^3^∙ha^−1^ water in each application. It was found that foliar nutrition with silicon, depending on the experimental combination, caused the increase of root yield by 7.5–25.1%, biological sugar yield by 7.1–23.2%, and commercial yield of sugar by 4.8–22.2%, compared to the control treatment (without silicon application). The largest increases in root yield, biological yield of sugar, and commercial yield of sugar were observed in the treatments with two and three applications of Actisil fertilizer, and this was significantly more than with a single application. In the case of other foliar fertilizers, the number of treatments did not have a significant impact on root yield, biological yield of sugar, and commercial yield of sugar. In most treatments, foliar nutrition with silicon did not significantly affect the sugar content in the roots, but increased the content of α-amino nitrogen. The effect of nutrition on the content of potassium was varied—either it did not have a significant impact or caused a significant increase. The sodium content did not substantially change due to application of foliar nutrition with silicon. The largest increase of yield by providing foliar nutrition with silicon was obtained in 2015, in which less favorable weather conditions for the growth and development of plants occurred. In the Czech Republic in the years 2014–2015, the effects of foliar nutrition of sugar beet with NanoFYT Si fertilizer (hydrated nanoparticles SiO_2_—230 g∙dm^−3^) and K-gel 175 (175 g K_2_O and 58 g S∙dm^−3^) were evaluated [12]. There were three treatments with foliar nutrition: (1) NanoFYT Si (0.5 dm^3^∙ha^−1^; 18 July) + NanoFYT Si (0.5 dm^3^∙ha^−1^; 19 August); (2) NanoFYT Si (0.5 dm^3^∙ha^−1^; 19 August), and (3) K-gel 175 (5 dm^3^∙ha^−1^; 6 August) + NanoFYT Si (0.5 dm^3^∙ha^−1^ 19 August), which were compared to the control treatment. The increase in root yield in the year 2014, which had favorable weather conditions for sugar beet growth, was 5.1% in the first treatment, 3.8% in the second treatment, and 4.2% in the third treatment compared to the control treatment (98.5 Mg∙ha^−1^). In the year 2015, which was characterized by a shortage in rainfall and higher air temperature, the increase in root yield was, respectively, 2.2%, 16.1%, and 24.5% in the treated plants (control treatment—71.8 Mg∙ha^−1^). In 2014, the yield of leaves decreased in the first treatment by 5.8%, in the second by 13.4%, and in the third by 1.7% as compared to the control treatment (36.1 Mg∙ha^−1^). In 2015, the plant response was slightly different: the yield of leaves in the first treatment decreased by 24.1%, in the second treatment increased by 1.6%, and in the third treatment by 21.5% (control treatment—24.7 Mg∙ha^−1^). The biological sugar yield in the years 2014–2015 increased on average in the first treatment by 6.4%, in the second treatment by 12.1%, and in the third by 19.6% in comparison with the control treatment (16.1 Mg∙ha^−1^). On the other hand, commercial sugar yield on average in the years 2014–2015 increased by 7.3%, 13.5%, and 21.2% respectively, in comparison with the control treatment (14.8 Mg∙ha^−1^). The sugar content in the roots increased on average in the first treatment by 3.14%, in the second treatment by 3.51%, and in the third treatment by 5.92% compared to the control treatment (19.1%). The content of α-amino nitrogen in the roots decreased in the first treatment by 16.7% and in the second and third treatments by 25.0% in comparison with the control treatment (30.0 mg∙100 g^−1^). The content of potassium in the roots increased in the first treatment by 4.0%, decreased in the second treatment by 1.7%, and in the third treatment by 13.9% compared to the control treatment (30.3 mmol∙kg^−1^). The sodium content in the roots decreased in the first treatment by 35.7%, in the second treatment by 48.6%, and in the third treatment by 54.4% compared to the control treatment (2.94 mmol∙kg^−1^). Similarly, the standard loss of molasses decreased by 0.81%, 17.1%, and 18.7%, respectively, compared to the control treatment (1.23%). The content of soluble ash in the roots increased in the first treatment by 0.74%, decreased in the second treatment by 7.13%, and in the third treatment by 9.34% in comparison with the control treatment (0.407%).

The increase in the yield of sugar of sugar beet caused by fertilization of silicon was caused by the increase in root yield, and this was the result of the formation of roots with a larger fresh weight.

## 6. Potato

In the study conducted on potato cultivation in Israel, effect of silicon fertilization was examined [36]. High silicon in tuber skin resulted in anatomical and compositional changes suggesting delayed skin maturation. Potatoes are susceptible to yield loss due to suboptimal growth conditions; thus, silicon fertilization may contribute to crop improvement. Compared to controls, skin cell area was greater, suberin biosynthetic genes were upregulated, and skin cell walls were enriched with oxidized aromatic moieties, suggesting enhanced lignification and suberization. Study of silicon metabolism in potato would contribute to ensuring food safety under changing climatic conditions.

In a study conducted in Brazil, silicon fertilization of the soil increased the average tuber weight, dry tuber weight, and tuber yield, especially in the absence of water deficit [13]. In Poland in the years 2009–2011, the effects of foliar nutrition with YaraVita Potato (P—192 g∙dm^−3^, K—62 g∙dm^−3^, Mg—40 g∙dm^−3^, Mn—10 g∙dm^−3^, Zn—5 g∙dm^−3^) and Actisil fertilizer (Si—6 g∙dm^−3^) on potato cv. Jelly were evaluated and compared to the control treatment (without foliar nutrition) [37]. Three treatments of foliar nutrition were examined: (1) YaraVita Potato: the first spray after canopy closure (2 dm^3^·ha^−1^), the second after 14 days from the first (10 dm^3^·ha^−1^), and the third after 14 days after the second treatment (10 dm^3^·ha^−1^); (2) Actisil: first spraying after plant emergence (0.3 dm^3^·ha^−1^), second treatment after 14 days from the first (0.4 dm^3^·ha^−1^), and third treatment 14 days after the second treatment (0.3 dm^3^·ha^−1^); and (3) YaraVita Potato + Actisil (both fertilizers were used in quantities and dates as in both previous treatments). Foliar nutrition did not have a significant impact on the yield of tubers. However, it had a significant impact on the reduction of the fraction of the smallest tubers (with a diameter below 30 mm) in a yield of approx. 50% and an increase in the share of the largest tubers (with a diameter of over 60 mm), especially after using Actisil fertilizer alone (+23%) or in combined with the fertilizer YaraVita Potato (+10%). Additional studies conducted in 2011 on the impact of the applied foliar fertilizers, YaraVita Potato and Actisil, on the quality of tubers such as darkening of the raw pulp and levels of phosphorus, potassium, magnesium, and calcium did not show any significant differences. However, it was found that the evaluated fertilizers significantly limited the accumulation of harmful nitrates in tubers, especially in treatments in which the Actisil silicon fertilizer was used together with YaraVita Potato—the nitrate content in this treatment was lower by almost 60%. Silicon fertilization had no significant effect on the quantity of the loss of natural potato tubers after 6 months of storage.

During cultivation, no significant differences were observed in the spreading of potato blight (*Phtytophtora infestans*) between treatments with foliar nutrition (regardless of the product used) and control treatment. A slightly slower development was observed on plants with foliar nutrition, but only in 2011, which is very favorable for the development of this disease. The applied foliar nutrition with Actisil fertilizer together with the chemical plant protection significantly inhibited the development of potato blight on the leaves during the growing season. The assessment of tubers after harvest in terms of disease infection was not unequivocal in each year of the study due to significant differences in the severity of individual diseases. It was observed that the use of Actisil fertilizer significantly limited the share of tubers with the symptoms of potato bacterial soft rot (*Erwinia carotovora* var. *carotovora*) with the simultaneous increase in the number of tubers with black scurf (*Rhizoctonia solani* spores). There was no significant effect of foliar fertilizers on the occurrence of hollow heart, internal heat necrosis, common scab (*Streptomyces scabies*), and dry rot (*Fusarium* spp.).

In studies conducted on three potato varieties in India in 2013–2017, the effects of using orthosilicic acid (Silixiol Potato) were studied [38]. After spraying seed potatoes with Silixiol Potato at a concentration of 1 cm^3^∙dm^−3^, the yield of tubers increased by 15%. After foliar application at a concentration of 4 cm^3^∙dm^−3^, the yield increase on average was 50%. However, if standard fertilizers with macroelements were applied, then after spraying the seed potatoes, no significant increase in yield was obtained, and after foliar spraying it was 20%. Based on these results, the authors argue that silicon plays a role in improving the use of nutrients by the plant. They also observed an improvement in the quality of tubers (reduction of tuber breaks, and hollow hearts). The cost–benefit ratio for the foliar application of orthosilicic acid (Silixiol Potato) was, on average, 1:6 for the farmer.

In Poland in 2011–2012, the effect of foliar nutrition in the form of Herbagreen Basic fertilizer with calcium and silicon on the yield and quality of early medium edible potato tubers cv. Finezja, recommended for the production of fries was examined [14]. Spraying was performed three times during the growing season: the first time in the initial growth period (at the potato plant height of 15–20 cm—the first decade of June), the second after about 2 weeks, and the third after about 4–5 weeks (the end of flowering growth stage). The effect was examined at different concentrations of mineral fertilization with nitrogen: 50, 75, and 100 kg N∙ha^−1^. In each application, a fertilizer concentration of 2.5 kg in 500 dm^3^ of water∙ha^−1^ was used. In unfavorable weather conditions, in 2011, a significantly higher yield increase of tubers after foliar application of Herbagreen Basic was obtained than in 2012, when the weather was more favorable. In 2011, after the foliar spraying, the total yield of tubers increased, on average, by 14.8%, and the commercial yield increased by 16.4% compared to the control object, whereas in 2012, the increments were much smaller and amounted to 5.5% and 7.7%, respectively, in relation to the control treatment. On average, the yield increase caused by foliar nutrition in the years 2011–2012 was 9% of total tuber yield and 11% of commercial yield. It was also shown that foliar nutrition with the tested fertilizer allowed one to limit the soil fertilization with mineral nitrogen. After application of 75 kg N∙ha^−1^ together with foliar nutrition, similar yield of tubers was obtained as at a dose of 100 kg N∙ha^−1^ without foliar nutrition, so the soil nitrogen dose could be reduced by about 25%, whereas in unfavorable weather conditions potato yield increased up to 50%. The foliar nutrition had a positive effect on the size of tubers, which was expressed by a larger, on average, 2%, increased share of tubers with a diameter of 50–60 mm and a 4% higher share of large tubers (diameter over 60 mm) in comparison with the control treatment. Assessment of the occurrence of external defects in tubers, i.e., deformation, greening, and common scab, showed a smaller percentage of their share in treatment with foliar nutrition, which resulted mainly from a smaller share of green tubers. Foliar nutrition did not have a significant impact on the content of starch, nitrates, and dry matter in tubers.

In India, using diatomaceous earth at a dose of 150 kg∙ha^−1^ with half the dose of the standard fertilizer (NPK + manure), the potato tuber yield was increased by 38.7% (24.3 Mg∙ha^−1^) compared to the control (17.5 Mg∙ha^−1^) and by 12.9% in relation to the full dose of standard fertilization (21.5 Mg∙ha^−1^) [15]. The use of diatomaceous earth in doses of 150, 300, and 600 kg∙ha^−1^, together with full standard fertilizer, resulted in the reduction of tuber yields in comparison with the same doses of diatomaceous earth, but with half the dose of standard fertilizer. This may be because of the higher extent of infection due to late blight in the later growing season. The application of diatomaceous earth at a dose of 600 kg∙ha^−1^ significantly improved the size of tubers and number of tubers weighing over 75 g (only full standard fertilizer) with 34 pcs per plot up to 64 pcs per plot. Foliar application of oligomeric silicic acid and boric acid in cultivation of potato in the Netherlands in 2003 increased the tuber yield by 6.5% and lowered disease-causing infection in plants [29].

In the study conducted in Iran, effect of four different silicon compounds (nanosilica, sodium silicate, nanoclay, and Bentonite) in two concentrations (1000 and 2000 ppm) on growth of potato plants was examined [39]. Silicon treatments, except sodium silicate, improved leaf properties (up to 18% in leaf dry weight in Bentonite (1000 ppm)) and increased stem diameter (up to 17% in nanoclay and Bentonite (1000 ppm)). All root traits were improved by the silicon fertilizer (up to 54% in root area per plant in sodium silicate (1000 ppm)). Although minituber production was not affected by silicon treatments, minituber quality characteristics were improved by silicon application in comparison with the control plants. 

The increase in potato yield caused by silicon fertilization was the result of the production of tubers with a larger fresh weight by plants. 

## 7. Meadows

The foliar application of the growth stimulator Optysil at doses of 46.8 g and 74.9 g Si∙ha^−1^ did not have a significant effect on the yield of dry matter of meadow vegetation compared to the control (without foliar nutrition with silicon) in studies conducted in Poland [40]. Increasing doses of silicon caused increase of yield of *Fabaceae* (red clover and white clover) and decrease in yield of other dicotyledonous plants. These authors found an increase of the nutritional value of meadow plants fertilized with silicon in relation to the control treatment. Feeding milking cows with silage from plants fertilized with silicon improved milk yield. The total solids, protein, and milk fat content was higher after using silicon. The highest milk yield, as well as protein, milk, and fat content, was obtained in the group of cows fed by silage from plants fertilized with a higher dose of silicon (74.9 g Si∙ha^−1^). The milk contained fewer microorganisms and somatic cells. The best results of this application were observed for milk produced by cows fed silage, with a variant having a smaller dose of silicon.

In the experiment including 60 temperate grassland species, positive species richness effected Si, as well as Ca stocks that were attributable to increased biomass production. The presence of particular functional groups was the most important factor explaining variation in aboveground Si and Ca stocks (mmol∙m^−2^). Grass presence increased the Si stocks by 140%, and legume presence increased the Ca stock by 230% [41].

Species composition of grasslands and pastures is an important control on biomass production and ecological functioning, with a significant role of grasses and legumes. Based on available literature, it is known that Si/Ca availability is an important trigger for shifts in abundance of plant families, especially legumes and grasses [42]. In the study, authors suggested an increase of meadow yield by Si due to better grass plant performance.

Improvement of nutritive value of meadow plants fertilized with silicon resulted from the increase in the share of Fabaceae plants while reducing the share of other dicotyledonous species.

## 8. Berry Crops and Vegetables

In the experiment conducted in Poland, it was observed that the use of Actisil as fertilizer at a concentration of 0.1% significantly improved the growth of strawberries, regardless of soil conditions [43]. Similar study conducted in Poland on foliar nutrition of strawberries with alkaline K + Si (49 g N-NH_2_, 360 g K_2_O, and 15 g SiO_2_∙dm^−3^) as a 1% solution applied twice (the beginning of plant flowering and just after flowering) did not affect the weight and diameter of the fruit. However, it significantly increased the dry matter content in fruit and its firmness, and was an important reason for the accumulation of a smaller amount of nitrates by the strawberry fruit [44]. In other studies conducted in Poland, an increase in firmness of strawberry fruit was found after application of Actisil as fertilizer [45]. At the Institute of Horticulture in Skierniewice (Poland), foliar application of Optysil in strawberry cv. Senga Sengana was used to protect against gray mold (*Botrytis cinerea*) and crown rot (*Phytophthora catorum*) [30]. The intensity of gray mold and crown rot on the strawberry on unsprayed strawberry plants was 10% and 14%, respectively. The severity of both diseases was lower in plots in which the fertilizer was applied at a dose of 0.5 and 1 dm^3^∙ha^−1^ four times from the beginning of flowering. Its efficiency ranged from 40 to 80% depending on the dose and time of evaluation, and was lower (first evaluation) or similar (second evaluation) to that obtained for standard fungicides cyprodinil + fludioxonil, pyraclostrobin + boscalid, and biological products based on *Pythium oligandrum*. The yield from plots treated with Optysil stimulator was significantly higher in comparison with those from control plots.

In Brazil, fertilization of crisp lettuce with silicon at a dose of 2.0 or 2.7 dm^3^·ha^−1^, on the 20th day after planting in the permanent place, increased its yield and postharvest firmness [46]. The aim of research conducted in the years 2009–2010 in Poland was to determine the impact of the application of silicon (0, 250, 500, or 750 mg·dm^−3^) and manganese (5.2 or 52.0 mg·dm^−3^) on the size and chemical composition of heads of lettuce cv. Omega F1 cultivated in a greenhouse [47]. Silicon was applied to the substrate once when putting on an aqueous solution of colloidal silica. The studies reported a significantly higher weight of heads and a higher dry matter content in plants fed with silicon at a dose of 250 mg·dm^−3^ compared to control treatment in which no silicon was used. In similar studies in Poland after application of Actisil as fertilizer, it was found that increasing silicon doses (0.21, 0.42, and 0.63 mg∙dm^−3^) significantly and positively affected the yield of lettuce grown in mineral wool using fertigation at a high content of manganese in the nutrient medium [48]. In studies conducted in Poland, the silicon supplied by fertigation to lettuce subjected to stress caused by the excess manganese significantly increased the fresh weight of the leaves, the relative water content (ratio of the current water content in the tissue to its content in fully hydrated tissue), and the number of leaves per plant, while reducing the dry matter content [49]. A significant increase in yield of lettuce leaves in relation to the control was obtained after using sea calcite (Herbagreen) in Serbia [50]. In the experiment conducted in Poland, spraying seed lettuce with 0.2% Alkaline K + Si (0.51% Si) resulted in a significant increase in the weight of 1000 seeds and their germination rate [51].

In Bulgaria in years 2011–2012, effect of fertilization with marine calcite using Herbagreen mineral fertilizer was examined. The effect of Herbagreen on seed production of cucumber, melon, and zucchini was evaluated. Three times treatment with the fertilizer at a dose of 0.04% at an interval of 14 days had a positive effect on seed yield in the studied cultures. Proven high effect was established in the indicator number of fruit per plant. There were no significant differences between treated and untreated (control) variants at the indicators: number of seeds per fruit, number of seeds per gram, weight of seeds per fruit, number of seeds per gram, or absolute mass of seeds. Increasing the yield of the seed does not have any negative effect on the quality of the seeds [52]. 

Studies with application of silicon to the soil conducted in China in the years 2005–2006 showed an increase in tomato yield by 8.7–15.9% (on average by 12.0%) compared with the control treatment [32]. The studies on tomato were conducted in Brazil with three silicon fertilizers (calcium silicate, potassium silicate, and sodium silicate) in five doses (0, 100, 200, 400, and 800 kg SiO_2_∙ha^−1^) [53]. Silicon fertilization increased the commercial yield of tomato and reduced the occurrence of cracked fruits. Si fertilization (401 kg∙ha^−1^ of SiO_2_) increased the yield of tomato to 60.8 Mg∙ha^−1^_._ Increase in economic returns by using silicon fertilization was significant because of higher productivity of tomato plants. 

In India, the largest yield of tomato was obtained using the full dose of the standard fertilizer (NPK) and diatomaceous earth at a dose of 750 and 500 kg∙ha^−1^ [54]. In the studies conducted in Poland, application of Actisil as fertilizer to tomato growing on rockwool mitigated manganese toxicity, which resulted in increased biomass production with lower content of manganese in the substrate (9.6 mg Mn∙dm^−3^, +8.2% for the cv. Alboney F1 and 16.8% for the cv. Emotion F1, with significant differences for Emotion F1) [55]. However, the use of a variant with a high content of manganese (19.2 mg Mn∙dm^−3^) did not affect the crop yield. In Greece, while growing tomatoes in hydroponics, it was found that fertilization with silicon increased fruit hardness, beta-carotene, and lycopene content, as well as dry matter and vitamin C content [56]. In Poland, fertilization with sodium silicate (Na_2_SiO_3_), calcium silicate (CaSiO_3_), or potassium silicate (K_2_SiO_3_) at a dose of 1.5 g·dm^−3^ substrate significantly increased the dry matter content in the tomato fruit extract but did not increase the yield [57]. The effect of the colloidal silica and three substrates with various silica contents and different capacities of orthosilicate monomers (mineral wool, sand, and straw) on the yield of greenhouse tomato was also investigated in experiments conducted in Poland [58]. Significantly higher total yield of fruits from plants fertigated with medium enriched in silicon (15.98 kg per plant) was observed compared to plants cultivated in control treatment. There were no significant differences in the total and commercial yield, as well as in the average weight of fruit from plants grown in mineral wool and straw. In studies conducted in Poland, the total yield of tomatoes from plants cultivated in sand was smaller compared to plants grown in mineral wool. Watering a tomato two times with 1% Silvit fertilizer solution at a single dose of 0.5 dm^3^ per plant had a positive effect on the yield of plants grown in a peat and compost substrate, which was in soil infected with soil pathogens [59]. The increase in commercial yield under the influence of silicon fertilization was caused by reduction of infection by corky root rot, which was found in large intensity in control plants, and significantly reduced their yield. 

In the study on onion in India, the highest yields were obtained using 200 kg Si∙ha^−1^ in the form of ash from bagasse [60]. In other studies conducted in India, the dose of 600 kg diatomaceous earth∙ha^−1^ as a source of silicon used with full fertilization (NPK + manure) allowed one to obtain the largest yield of onion [61]. The enrichment of the peat substrate with calcium silicate (CaSiO_3_) or ammonium silicate ((NH_4_)_2_SiO_3_) increased the weight of the onion seedlings by more than 35% in studies conducted in Poland [62]. Foliar application of oligomeric silicic acid and boric acid in onion in the Netherlands in 2003 increased the yield by 10.8% and improved the uniformity of the yield [29]. In investigations in which silicon was applied to the soil conducted in north-eastern China in the years 2005–2006, cucumber yields grew by 9.35–26.6% (on average by 13.7%) in comparison with the control treatment [32]. In Poland, the studies evaluated the effect of using sodium (Na_2_SiO_3_), potassium (K_2_SiO_3_), calcium (CaSiO_3_), and ammonium ((NH_4_)_2_SiO_3_) silicates added to peat substrate on cucumber yield [63]. Positive effect of the added calcium and ammonium silicates on crop yield was found (a significant increase in the yield was obtained using ammonium silicate). Effect of other silicates was not unequivocal and depended on the concentration of fertilizers. The increase in cucumber yield was associated with an increase in the number of fruits, not their weight. In the study conducted in Poland, significant increase in the yield of cucumber fruit was obtained by the providing nutrition to roots of plants with silicon in the form of a silicate sol and using two substrates with a different content of silica at a dose of 500 and 750 mg Si∙dm^−3^ in comparison to plants from the control treatment [64]. In a study with peppers conducted in India in 2015–2016, foliar application of silicon (potassium silicate containing 18% Si) with zinc (0.2%) and boron (0.1%), regardless of the concentration of silicon in spraying (0.1, 0.2, and 0.4%), significantly increased plant height and biomass production both 45 days after planting and during fruit maturity [65]. The highest yield was obtained using a concentration of 0.4% Si.

Better quality of strawberries after silicon fertilization was the result of improving their health and firmness. The increase in the yield of lettuce was caused by the greater fresh weight of the heads. In the case of tomatoes, silicon increased yield of fruit from the plant, better healthiness, and hardness. The increase in cucumber yield due to the use of silicon was caused by the increase in the number of fruits per plant.

## 9. Orchards

At the Institute of Horticulture in Skierniewice (Poland), Optysil was applied foliarly to the cv. Golden Delicious apple tree to provide protection against the apple scab (*Venturia inaequalis*) [30]. Field trials were conducted in 2013 when weather conditions were very favorable for the development and spread of diseases. The intensity of apple scab on unprotected trees reached 40% of leaves in early June, and in July it reached 82% of leaves and 60% of fruits. The efficacy of Optysil 0.5 and 1 dm^3^∙ha^−1^ applied six times from the “pink bud” stage (BBCH 57) ranged from 67% to 81% and from 78% to 80%, respectively, in the case of scab on leaves and fruits, and it was similar (on the leaves) or lower (on the fruit) when compared with standard plant protection using fungicides captan and dithianon. Foliar application of oligomeric silicic acid and boric acid in orchards in the Netherlands in 2005–2008 resulted in a 17% increase in yield, improved uniformity, and Brix value [29]. In Poland, application of the Actisil fertilizer caused improvement of the quality of the sweet cherry fruits, which was obtained partly by reducing their cracking [66].

In China, silicon fertilization at a dose of 60 kg SiO_2_∙ha^−1^ (two types of slag containing silicon) increased the yield of grapes by 13.5% compared to the control treatment (fertilization without silicon) and improved their quality [67]. It also prolonged the fruit’s firmness. There were also beneficial effects of fertilization with marine calcite (Herbagreen) fertilizer in viticulture in experiments conducted in Turkey [68].

The purpose of the study conducted in Poland was to assess the influence of foliar fertilization with Mn, “Alkalin” (N, K, and Si fertilizer), and Mn + “Alkalin” on physical features and chemical composition of chokeberries. Fertilization with “Alkalin” significantly increased firmness of fruit in comparison to control and other treatments. However, neither Mn nor “Alkalin”, nor the combined fertilization, increased total sugar content in chokeberries. On the other hand, fruits of the combined treatment showed the highest saccharose content [69].

Increase in yield and improvement of fruit quality resulting from the use of silicon were caused by the increase of their fresh weight and better their healthiness. 

## 10. Ornamental Plants

The effect of Actisil on the growth and flowering of plants *Gazania rigens* (L.) Gaertn., *Salvia farinacea* Benth., and *Verben hybrid* Voss., commonly grown in flower beds and on balconies, was investigated in Poland [70]. Silica solutions at concentrations of 120 and 240 mg Si∙dm^−3^ were used in the experiment and compared to the control (no spray). The spraying was performed two, four, and six times in weekly intervals. The beneficial effect of the fertilizer on the majority of morphological features of the tested species evaluated at the beginning of flowering of plants was demonstrated. The best effect on plants was observed for two or four treatments with a silicon solution of 120 mg Si∙dm^−3^. In another experiment in Poland, the pansies of the Fancy type sprayed with Actisil fertilizer, regardless of concentration, had dark green, rigid, and firm rosettes [71]. Actisil fertilizer at a concentration of 0.2% improved the appearance of the pansies. The plants were low and branchy. The stimulating effect of silicon in Actisil was also observed in Poland in the cultivation of chrysanthemums [72]. Plants sprayed with Actisil were healthy, homogenous, and properly formed.

The beneficial effect of applying silicon on ornamental plants resulted from the improvement of their health, firmness, and proper flower formation. 

## 11. Conclusions

The presented research results confirm, in the vast majority, the beneficial effect of silicon applied to soil or as a foliar application on the quantity and quality of the yield of many plant species. The effect of silicon as foliar nutrition, which stimulates plants to grow under stress conditions, is particularly beneficial. Such application should be introduced into plant production as a standard in the cultivation of crops.

In the near future, it is advisable to conduct further research that will allow for the determination of optimal times for the application of foliar nutrition and silicon doses in specific crops in various soil and climatic conditions. The introduction of such applications may improve the competitiveness of agricultural products from Europe.
